# Significance of *Artemisia Vulgaris* L. (Common Mugwort) in the History of Medicine and Its Possible Contemporary Applications Substantiated by Phytochemical and Pharmacological Studies

**DOI:** 10.3390/molecules25194415

**Published:** 2020-09-25

**Authors:** Halina Ekiert, Joanna Pajor, Paweł Klin, Agnieszka Rzepiela, Halina Ślesak, Agnieszka Szopa

**Affiliations:** 1 Chair and Department of Pharmaceutical Botany, Medical College, Jagiellonian University, Medyczna 9, 30-688 Kraków, Poland; asiek.pajor@student.uj.edu.pl; 2 Family Medicine Clinic, Medizinisches Versorgungszentrum (MVZ) Burgbernheim GmbH, Gruene Baumgasse 2, 91593 Burgbernheim, Germany; bag-burgbernheim@gmx.de; 3 Museum of Pharmacy, Medical College, Jagiellonian University, Floriańska 25, 31-019 Kraków, Poland; agnieszka.rzepiela@uj.edu.pl; 4 Department of Plant Cytology and Embryology, Institute of Botany, Faculty of Biology, Jagiellonian University in Kraków, Gronostajowa 9, 30-387 Kraków, Poland; halina.slesak@uj.edu.pl

**Keywords:** medical history, traditional medicine, chemical composition, biological activity, safety of use

## Abstract

*Artemisia vulgaris* L. (common mugwort) is a species with great importance in the history of medicine and was called the “mother of herbs” in the Middle Ages. It is a common herbaceous plant that exhibits high morphological and phytochemical variability depending on the location where it occurs. This species is well known almost all over the world. Its herb—*Artemisiae vulgaris herba*—is used as a raw material due to the presence of essential oil, flavonoids, and sesquiterpenoids lactones and their associated biological activities. The European Pharmacopoeia has listed this species as a potential homeopathic raw material. Moreover, this species has been used in traditional Chinese, Hindu, and European medicine to regulate the functioning of the gastrointestinal system and treat various gynecological diseases. The general aim of this review was to analyze the progress of phytochemical and pharmacological as well as professional scientific studies focusing on *A. vulgaris*. Thus far, numerous authors have confirmed the beneficial properties of *A. vulgaris* herb extracts, including their antioxidant, hepatoprotective, antispasmolytic, antinociceptive, estrogenic, cytotoxic, antibacterial, and antifungal effects. In addition, several works have reviewed the use of this species in the production of cosmetics and its role as a valuable spice in the food industry. Furthermore, biotechnological micropropagation of *A. vulgaris* has been analyzed.

## 1. Introduction

In 2015, the awarding of the Nobel Prize in Medicine for the discovery of artemisinin, a compound of plant origin found in *Artemisia annua* (annual mugwort), inspired the researchers to study the phytochemical and pharmacological properties of other species of the genus *Artemisia*. Recently, this species has been taken under consideration to be active toward the virus SARS-CoV-2 and disease COVID-19 [1,2].

*Artemisia vulgaris* L. (common mugwort) is one of the best-known species of this genus, which has a widespread distribution in the natural habitats worldwide (Europe, Asia, North and South America, and Africa). For many centuries, this species has been mainly used for treating gynecological ailments and gastrointestinal diseases [3,4,5,6,7]. Recently, researches have proved that this species exhibits antioxidant, hypolipidemic, hepatoprotective, antispasmolytic, analgesic, estrogenic, cytotoxic, antibacterial, antifungal, hypotensive, and broncholytic effects [8,9,10,11,12,13,14,15,16,17].

The different applications of this plant species have been possible due to its rich chemical composition, which especially includes essential oils, flavonoids, sesquiterpene lactones, phenolic acids, coumarins, and other groups of metabolites.

The presence of essential oil in *A. vulgaris* contributes to the significance of this species as a culinary spice in the food industry in various regions of the world. Currently, this species is also increasingly used in the production of cosmetics in Europe as well as in Asia and North America [14,18,19,20].

The latest review on *A. vulgaris* presented by Brazilian–Iran and Malaysian teams highlighted the value of this plant species from the South American–Asian point of view [21].

The present review provides classical information about the importance of *A. vulgaris* in therapeutics and the food industry from the European point of view and additionally discusses the possible new applications of this plant species in phytotherapy as a hepatoprotective, broncholytic, anthelmintic, and cytotoxic agent and in the cosmetics industry as a raw material in Europe, East Asia (especially in Korea), and North America. 

## 2. Distribution and Taxonomic Consideration of *Artemisia*

The *Artemisia* genus belongs to subtribe Artemisiinae of tribe Anthemidae from Asteraceae family and comprises more than 500 species. These species are distributed all over the world, especially in the moderate climate zones of Europe, East Asia, Americas, North Africa, and Australia [22,23,24].

The plants of this genus are annual, biennial, and perennial herbs or small shrubs and halfshrubs. From the chemotaxonomic point of view, these plants are rich in essential oils and bitter substances (particularly sesquiterpenoid lactones). They are also a good source of flavonoids, coumarins, and phenolic acids [25].

Several representatives of this genus are found in Asia—about 150 species in China and about 174 in the ex-USSR. Many species are characteristic of Japan and Iran flora, (about 50 and 35, respectively). The number of *Artemisia* sp. occurring in Europe is estimated at 57 [26].

In Poland, 10 different species, including *A. vulgaris* and *Artemisia absinthium*, are found in the natural habitats [27]. Some of them (e.g., *Artemisia petrosa* Baung and *Artemisia pontica* L.) are distributed only on small areas in the country and are on the list of endangered plant species [27,28].

One of the most popular species worldwide is *A. vulgaris* (common mugwort). It is known by various synonymous Latin names, presented in Table 1. This can be attributed to the broad distribution of this species on all continents and the differences in its chemical and genetic composition, which is characteristic of the plants of different origins.

According to the data available on the official website “The Plant List” (created by Global Strategy for Plant Conservation and World Flora Online), *A. vulgaris* has as many as 107 synonymous Latin names, including *Absinthium spicatum* (Wulfen ex Jacq.) Baumg [3,29], *Artemisia affinis* Hassk. [3,29], *Artemisia opulenta* Pamp. [3,29,30], *Artemisia vulgaris* subsp. *vulgaris* [31], and *Artemisia vulgaris* var. *indica* (Willd.) Hassk. [3,29,31] (Table 1). In addition, again due to its widespread dissemination, this species is known by various foreign-language names, including Carline Thistle, Chiu Ts’Ao, common mugwort, Chrysanthemum weed, Cingulum Sancti Johannis, common wormwood (English), Ajenjo, altamis, altamisa (Spanish), altamiza, amarella (Italian), armoise, Armoise citronnelle (French), beiai (Chinese), Beifußkraut (German), Nagadamani (Ayurvedic), moxa (Japan) and Polynesian snare (Russian) [3,4,29,30,31].

## 3. Genetic Issues

*Artemisia* (Asteraceae, Anthemideae, Artemisiinae) is the largest genus of the tribe Anthemideae. It is also one of the broadest genera of the family Asteraceae, which comprises more than 500 species [32,33]. The genus exhibits high morphological and phytochemical variability and ecological plasticity, with species occurring from sea level to huge mountains and from arid zones to wetlands [22]. At present, the following five main groups are considered at the subgeneric level, mostly based on the floral characteristics: *Artemisia*, *Absinthium* (Mill.) Less., *Dracunculus* (Besser) Rydb., *Seriphidium* Besser, and *Tridentatae* (Rydb.) McArthur [33,34].

According to Reference [32], *Artemisia* has two basic chromosome numbers, the largely predominating *x* = 9 and the less extended *x* = 8, with the ploidy levels ranging from diploid to dodecaploid in the species with *x* = 9 chromosomes and from diploid to hexaploid in those with *x* = 8 chromosomes. The chromosome number *x* = 9 is the most common not only in the genus *Artemisia* but also in the tribe Anthemideae and the family Asteraceae [33]. The evolutionary pathway determining the chromosome number in *Artemisia* is affected by the successive or simultaneous effects of polyploidy (both autopolyploidy and allopolyploidy) and dysploidy [35]. The existence of two basic chromosome numbers indicates that *Artemisia* is a dysploid genus. Dysploidy is seen in *Artemisia*, *Absinthium*, and *Dracunculus*, which are the three main groups of the genus, while *Seriphidium* and *Tridentatae* display only one basic chromosome number, *x* = 9 [32].

According to Reference [34], dysploid–polyploid complexes have played a major role in the karyological evolution of *Artemisia*. Dysploidy refers to change in the chromosomal base number through the rearrangement of chromatin and the loss or gain of a centromere without any change in the amount of chromatin in the karyotype. A high percentage of *Artemisia* species are polyploids, and the reported cytotypes cytogenetically differ in their external morphology, anatomy, fertility, and phytochemical characteristics [35]. Among the species, *A. vulgaris* is quite diverse showing several cytotypes, of which 2*n* = 2*x* = 18 is the most common [36].

In most cases, differences in the 2C values of about sevenfold have been detected between the species. These are directly related to the polyploidization events [33], which reflects the high genetic plasticity of the genus *Artemisia*. This phenomenon is also seen in all the major groups of this genus. In diploids, the chromosome number is most often 2*n* = 18 or 16, but ploidy levels of up to 16× have been reported. The highest chromosome number known is 144, and aneuploidy is frequent as well [37].

In Europe and North America, as well as in Altai and Russia, the most commonly observed ploidy of *A. vulgaris* was 2*n* = 16 (*x* = 8) [38], while specimens from the high Himalayas, which underwent a period of glaciation, were found to be mainly diploids having 2*n* = 18 chromosomes [39]. At different elevations in this same region, examples of tetraploid (2*n* = 36) and hexaploid (2*n* = 54) were also found [39]. However, biotypes from other regions of the world have been reported with chromosome numbers of 2*n* = 16, 18, 24, or 36 [7].

*Artemisia* is one of the 530 genera of the Asteraceae family in which polyploidy was detected. It is a good example of polyploid series and ploidy-level variation, with both autopolyploids and allopolyploids reported [35]. According to Reference [33], approximately 43.5% of *Artemisia* species are exclusively diploid and 29.7% are exclusively polyploid, while around 26.8% of species are both diploid and polyploid. The high percentage of polyploids and the ability of the species to colonize different ecosystems, as well as their adaptation to different habitat conditions, may be the main features that favored the successful expansion of this genus across the landscape.

The chromosomes of *Artemisia* are rather small (2–8 μm). Their karyotypes tend to be symmetric, considering both the interchromosomal and intrachromosomal asymmetry [33,39]. The use of Giemsa C-banding and chromomycin and bisbenzimide fluorochrome banding allowed using the heterochromatic chromosome segment banding patterns for systematic study and characterizing *Artemisia* as a genus having few intercalary C-bands in the chromosomes [33,39].

The distribution of heterochromatin varies between taxa. The population of *A. vulgaris* is characterized by centromeric or pericentromeric heterochromatin. The cytogenetic data, especially those related to the heterochromatin, may be helpful in understanding the role played by polyploidy in the evolution of the genus *Artemisia*. The diploid and tetraploid taxa were identified to have different chromosome structures: the tetraploid species studied showed two types of heterochromatin (AT- and CG-rich), whereas the diploids showed only the GC-rich regions [40]. 

The molecular phylogenetic data basically agree with the classical taxonomy, but some questions remain unsolved. The taxonomic classification of *Artemisia* sp. has been controversial due to the following reasons: insufficient diagnostic features, highly variable morphological traits, the possibility of natural hybridization among taxa, polyploidy, and nomenclatural legacy [35].

According to Reference [41], sequencing of nuclear and organelle genome regions, such as the external and internal transcribed spacers of nuclear ribosomal DNA and the intergenic spacers between the genes of chloroplast genome (plastome), has enabled the researchers to perform molecular phylogenetic analyses of *Artemisia*. They reported that the complete plastome sequences of this genus are sufficiently polymorphic to be used as superbarcodes for its species. This in turn will facilitate the development of new molecular markers and allow studying the phylogenomic relationships of *Artemisia* species in the family Asteraceae.

## 4. Botanical Characteristics

The species *A. vulgaris* shows high morphological variability depending on the place of occurrence [30]. Comparative studies conducted between different populations revealed variability in the branching (presence or absence and extent), leaf shape, and root diameter of the plant [7]. The high variability of *A. vulgaris* has been confirmed by the study conducted by Barney and DiTommaso on two populations collected from different, geographically isolated areas of Ithaca (USA). The authors found that plants from one population had densely hairy stems and light green leaves, each with a few deep notches, whereas those collected from another part of the city had almost smooth stems and dark green leaves with many deep notches [42].

*Artemisia vulgaris* is a herbaceous plant which grows up to a length of 2.5 m and has a width of 75 cm. It is characterized by an intense aroma that is readily released when the leaves are crushed [30,43], and a spicy taste [44]. The plant has a thick main root and many small, fibrous lateral roots. The roots take on a light-brown color and measure up to 1 cm. They remain in the upper layer of the soil, at a depth of 7–18 cm, forming a vast, underground network [7,30]. The stems of the plant are slightly wavy, straight, or branched, having a brown color at the lower end, and become woody with age, appearing green further up and purple at the top. Some of the stems are also hairy [7,45]. The leaves are 5–10 cm long. They are set densely, and alternately, primarily in the upper parts of the stem. The lower leaves with short petioles are divided into segments and take on a feathery shape, while the middle and upper ones are smaller and single or double pinnate. The dorsal side of the leaves has a dark green color, while the ventral side is whitish and tomentose [7,46]. Small, almost bare, yellowish or brown-red flowers are embedded in small baskets that form heavily branched panicles with numerous lanceolate bracts at the top of the shoots [46]. One basket may contain around 15–30 flowers with numerous stamens [7]. Studies conducted in the eastern part of the USA showed that the inflorescences contain 52% of ligulate flowers and 48% of tubular flowers [7], of which 25–50% are female [47].

After the flowering period, which lasts from July to September in Europe, *A. vulgaris* develops fruit—called achenes. The seeds of these fruits are brown, weighing 0.12–0.14 mg, and have a narrow base. They are oblong and ridged, covered with fine hairs on the apex [7,48].

The species reproduces from seeds, which can produce up to 200,000 per year depending on the habitat [49], but so far this has been observed only in the native places of occurrence, namely in Asia and Europe. Some biotypes of this plant do not produce reproductive seeds [5]. The seeds of *A. vulgaris* spread through wind, beetles, and flies [47]. The plant usually reproduces vegetatively with the help of its roots, which can survive in the ground during winter. In the northern USA, pieces of roots are often moved long distances by local floods [7].

The leaves and roots of *A. vulgaris* exhibit strong allelopathic properties [42,50]. Although this has been confirmed in fresh leaves, it was not possible to isolate the specific compound that is responsible for these properties. It is suspected that these are contributed by a mixture of compounds from the monoterpenoid group [42].

## 5. Natural Habitats and Cultivation

Most sources indicate that *A. vulgaris* originated from Europe and Asia [7,48], and from there, this species was brought to North America, probably at the beginning of the 16th century [7].

At present, the plant is abundantly seen in many regions of the world, ranging from the Himalayas in Asia, through Europe, to the warm areas of North America [22,30]. The only continent where *A. vulgaris* does not occur is Antarctica [7].

This species is widely considered a weed [7,31]. It can be found in many habitats, for example, on roadsides, along rivers, or in abandoned mines, thickets, tree nurseries, and arable or other fields, where it interferes with the growth of different plants [3,5,7].

Individual populations of *A. vulgaris* are well adapted to live in a wide range of pH and various soil types, including sandy and loamy. Due to its extensive root system, this plant can quickly occupy large areas [7]. Controlling the spread of *A. vulgaris* is very difficult because only a few effective ways can limit its growth [5,7].

As a species with low requirements, *A. vulgaris* can easily colonize successive sites and displace native species. Thus, it can easily disturb the local ecosystems [7,30].

This species is cultivated on an industrial scale in Italy, France, Brazil, and Japan, as well as in the mountainous regions of India and Sri Lanka [3]. It is also possible to grow this plant in home gardens or can be obtained from natural habitats.

The aerial parts of the plant die each year [46], and hence, they are harvested at the beginning of flowering. The parts are obtained by cutting the tops of shoots, while the woody stems are omitted. Then, these are dried in airy drying sheds under natural conditions [18]. After drying, the herb has a spicy, bitter taste and a balsamic aroma.

The appropriate time to harvest the roots is at the beginning of winter [18]. Drying is carried out at 40 °C in drying sheds. The roots that are properly harvested and dried are brittle and have a light-brown color.

## 6. Status of the Plant in Official Phytotherapy in Europe

The aerial parts of *A. vulgaris*—*Artemisiae vulgaris herba*—are usually used as a pharmaceutical raw material [31], while the roots—*Artemisiae vulgaris radix*—harvested in early winter, are less frequently used [18]. Both these raw materials do not have their monographs in the latest editions of pharmacopoeias; however, *A. vulgaris herba* had a monograph in the German Pharmacopoeia published in 1988 [51]. In the latest European Pharmacopoeia [52] and in the French Pharmacopoeia [53], the species is only mentioned as a homeopathic raw material. Although the plant has a monograph published by the EFSA [18], no opinions regarding it have been issued by the European Scientific Cooperative on Phytotherapy (ESCOP) or European Medicines Agency (EMA).

## 7. Phytochemical Characteristics

**Figure 1:**
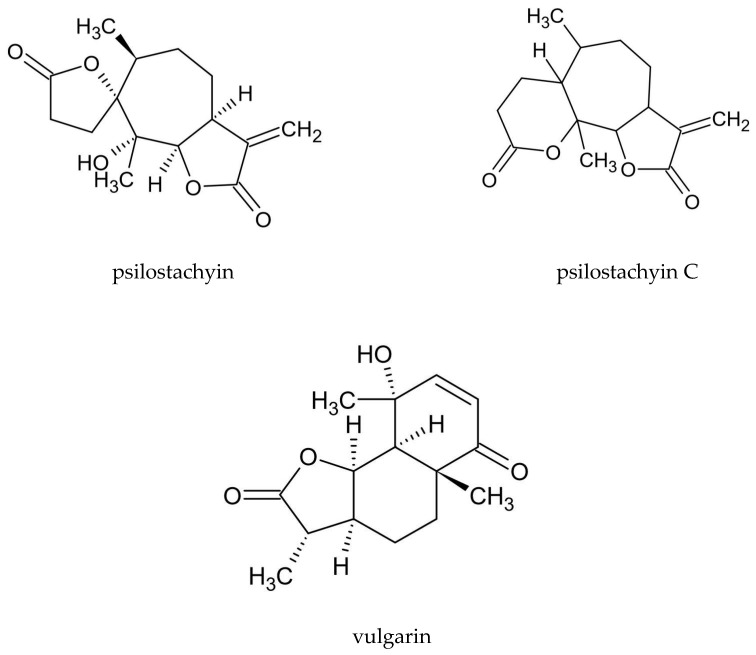
The chemical structure of sesquiterpenoid lactones characteristic of *A. vulgaris.*

Various groups of compounds can be distinguished in *A. vulgaris*, including sesquiterpenoid lactones, flavonoids, coumarins, phenolic acids, sterols, polyacetylenes, carotenoids, vitamins, and cyanogenic glycosides. Essential oil is another important substance found in the plant. Due to the high intraspecific diversity and discrepancies in the chemical composition of the plant determined by using various test methods, it is difficult to indicate a distinct phytochemical profile for *A. vulgaris* [15]. A characteristic feature of this species is the presence of sesquiterpenoid lactones, including psilostachyin, psilostachyin C, and vulgarin (Figure 1), and also artemisinin was confirmed. In addition, the presence of flavonoids—derivatives of kaempferol and quercetin, and coumarin compounds, such as esculin, umbelliferone, and scopoletin, is a distinguishing attribute of the plant (Table 2).

**Figure 2:**
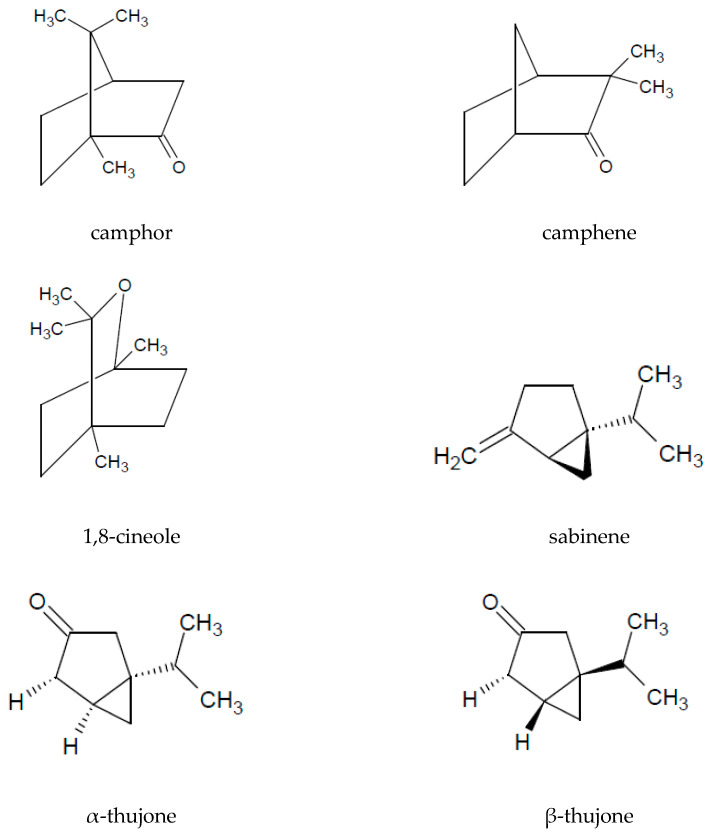

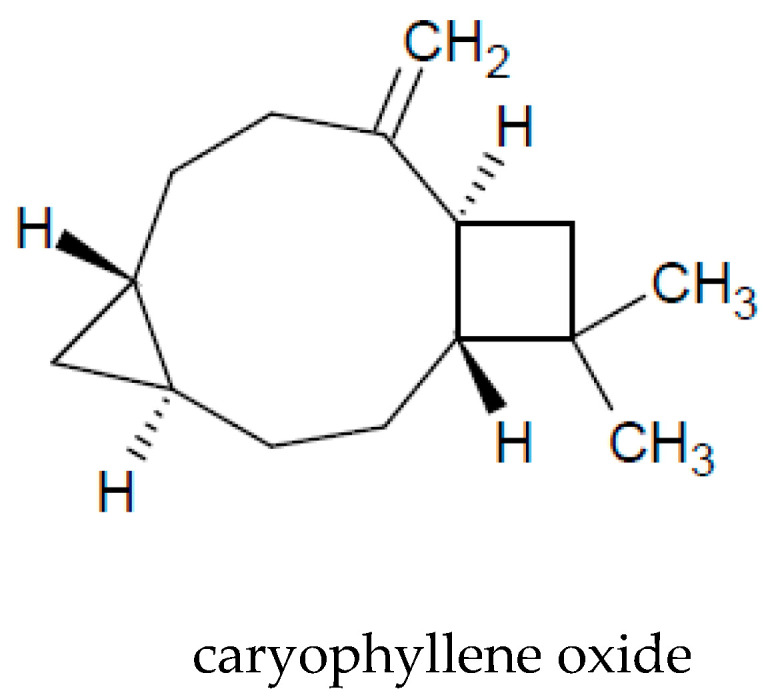
The chemical structure of volatile compounds characteristic of the essential oil of *A. vulgaris* herb.

The presence of essential oil is an interesting characteristic of *A. vulgaris*. A major part of the oil extracted from the aerial parts is constituted by monoterpenoids (72%) and sesquiterpenoids (26%). Among the volatile compounds, the following are the most commonly identified: 1,8-cineole, sabinene, camphor, camphene, caryophyllene oxide, α-thujone, and β-thujone (Table 3 and Figure 2).

Many experiments have revealed the significant differences in the quality and quantity of the components of the essential oils obtained from the *A. vulgaris* plants grown in different parts of the world (Table 4).

**Figure 3:**
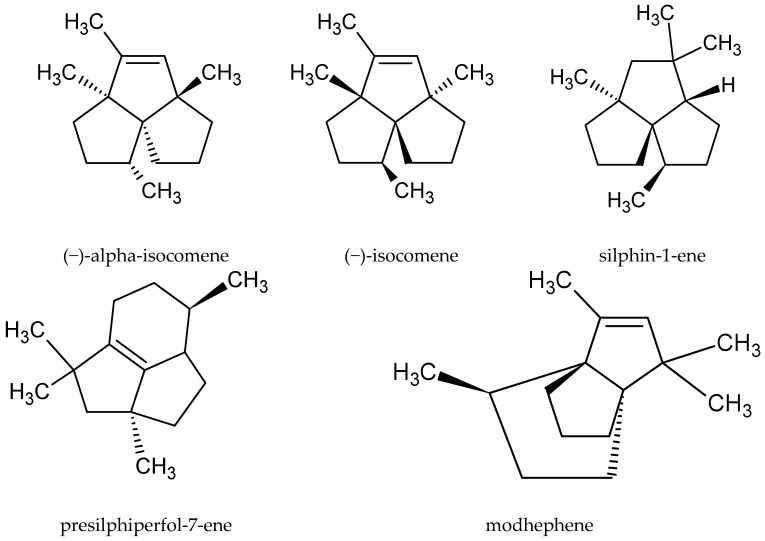
Chemical structure of the tricyclic sesquiterpenoids characteristic of the root of *A. vulgaris.*

Although the root of this species is sometimes used as raw material, only a few publications have described its chemical composition. A group of scientists from the University of Niš in Niš (Serbia and Montenegro) analyzed the differences in the phytochemical profile of the essential oil obtained from its green parts and root. The plant materials used in the study were collected during the flowering period. They were dried and then subjected to steam distillation in a Clevenger apparatus. The isolated compounds were identified using gas chromatography, gas chromatography–mass spectrometry, and ^13^C nuclear magnetic resonance. The main compounds in the oil extracted from the root were neryl 2-methylbutanoate (13.2%), β-eudesmol (10.4%), and bornyl 3-methylbutanoate (8.45%). However, none of these substances was found in the oil obtained from the aerial parts. The underground part of the plant also contained some rare compounds belonging to, *inter alia*, the group of tricyclic sesquiterpenoids, such as (−)-α-isocomene, (−)-isocomene, silphin-1-ene, presilphiperfol-7-ene, and modhephene [16] (Figure 3).

In the essential oil extracted from the aerial parts of *A. vulgaris*, no tricyclic sesquiterpenoids were detected. The dominant compounds that were identified in the study were 1,8-cineole (28.9%), sabinene (13.7%), β-thujone (13.5%), and β-caryophyllene oxide (6.5%), but these were absent in the underground parts of the plant. Based on these findings, the authors concluded that the differences in the chemical composition of the oils extracted from the root and aerial parts might indicate the coexistence of different biogenetic pathways in the plant species [16].

## 8. History of Medicinal Use

Due to its widespread occurrence, *A. vulgaris* was well known in ancient Egypt, Greece, and Rome. According to ancient belief, its name is derived from the name of the Greek goddess Artemis, who is the patron of pregnant women and newly delivered mothers. Because of its beneficial effects on menstruation- and pregnancy-related ailments, *A. vulgaris* had great importance in the religious rites devoted to the goddesses Isis, Artemis, and Diana. The healing properties of this species were described in medical works in as early as the 1st century A.D. by Dioscorides in “*Materia medica*” [81], by Pliny the Elder in “*Naturalis Historia*” [82], and by Galen in “*De simplicium medicamentorum facultatibus*” [83]. Furthermore, the mugwort plant was credited with warming and drying effects, and therefore, it was also recommended for the treatment of urological diseases, such as dysuria or nephrolithiasis. In medieval medicine, *A. vulgaris*, called “*mater herbarum*” (the mother of herbs), was used externally for treating wounds, against gout, and to remove leg fatigue, as well as in an attempt to treat fever [84]. In addition, the plant gained popularity as a remedy for gastrointestinal ailments “resulting from cold” [85], including stomach pain, diarrhea, and intestinal colic. It was believed to be effective against jaundice when served with wine and against goiter when applied as a poultice [86]. During the Renaissance, thanks to, *inter alia*, the invention of printing by J. Gutenberg, the holistic medicine flourished in Europe, lasting until the 18th century. At that time, in addition to women’s diseases, the therapeutic spectrum of *A. vulgaris* was expanded to include spleen and liver diseases. Additional recommendations appeared “for enlarged and distended spleen” [87], “against clogged liver” [88], and “for cold lower abdomen” [89]. During the development of modern medicine in the 19th century, epilepsy and neurosis were included among the indications for treatment with mugwort [90]. In the 20th century, the scientific and laboratory analyses of the composition of *A. vulgaris* led to the declaration that due to its high allergic potential the herb is not suitable for medicinal use and can only be used in culinary applications, and that its place in the household is the kitchen, not the medicine cabinet [63].

## 9. Applications in Traditional Medicine Worldwide

In Asian medicine, *A. vulgaris* is often used for alleviating gastrointestinal discomfort and treating gynecological diseases [5,6].

In China, *A. vulgaris* is traditionally used to treat cholera and leprosy. Other indications include hemorrhagic conditions, such as the presence of blood in sputum, stool, and vomit, and nosebleeds [18]. The essential oil of this species is used as a popular herbal medicine called “Ai Hao,” and is prescribed for curing ulcers and diarrhea [7].

The Traditional Chinese Medicine (TCM) also recommends thermopuncture consisting in burning “moxa”—dried and powdered leaves of *A. vulgaris*—directly on or close to the skin, or inhaling the resulting smoke, which allows the migration of “chi” energy [91]. Indications for this treatment include tumors, although its effectiveness has not been confirmed so far [92]. In 2009, Reference [93] investigated the harmfulness of compounds formed during the combustion of this plant species. The authors tested the smoke emitted by moxa for the presence of compounds commonly found in cigarettes, such as tar, nicotine, carbon monoxide, polycyclic aromatic hydrocarbons, ammonia, hydrogen cyanide, polyaromatic amines, volatile organic compounds, semi-volatile compounds, phenols, and carbonyls (e.g., formaldehyde and acetone), all of which are associated with the highest risk of cancer. Their results showed that even at concentrations higher by an order of magnitude than that achieved with the inhalation of normal smoke, only a few of the analytes exceeded the permissible standards. These included aromatic amines such as 1-aminonaphthalene, 2-aminonaphthalene, 3-aminobiphenyl, and 4-aminobiphenyl. Therefore, the authors concluded that burning moxa should not pose a health risk. Evidence of exceeded limits appeared only after the extrapolation of results for long-term use, which probably did not reflect the actual concentration of the tested compounds [93]. In TCM, *A. vulgaris* is most often used in thermopuncture in the form of cotton buds impregnated with the plant extract, or as cigarettes filled with dried leaves and used to cauterize the skin.

The species is also considered a substitute for cannabis. When being smoked, it exhibits mild intoxicating properties and strong relaxing properties [18]. In traditional Hindu medicine (Unani), many preparations based on *A. vulgaris* are used. For instance, “Arq-e-Afsanteen” is a preparation recommended for liver inflammation and obstruction. “Dava-ul-Luk” is used for treating enlarged liver or spleen and nephrolithiasis. The medication “Qurs-e-Gul” is indicated in chronic fever as a liver tonic, while decoctions from the plant are taken during dysmenorrhea [94]. In addition, liquid extract or dried greens at a single dose of 0.5–2 g is used in Ayurvedic medicine [3].

The species is also well known in the traditional medicine of South America, and often used against fever, malaria, and gastric disorders [95,96,97].

In European folk medicine, after oral administration, the *A. vulgaris* herb stimulates the secretion of gastric juice. Hence, it is used against gastrointestinal catarrh, insufficient production of bile and digestive juices, flatulence, and poor appetite. The plant is also used as a relaxant for the gastrointestinal tract and bile ducts and for relieving colic [3], while the observed laxative effect is utilized in the treatment of obesity. It is also used as infusions for external use for alleviating rheumatic and leg pains, as well as for preparing sitz baths for hemorrhoids. Other traditional applications of *A. vulgaris* include the treatment of nervous system disorders such as insomnia [31], epilepsy, depression, and excessive stress exposure [69]. Furthermore, it is recommended for relieving hypertension and inducing labor or miscarriage [98].

## 10. Applications in Modern Phytotherapy and Position in Official European Medicine

At present, *A. vulgaris* herb is not commonly used as a medication. However, due to its aroma and bitter taste, the herb is used for stimulating the secretion of digestive juices in the treatment of appetite loss, achlorhydria, gastritis, and flatulence [3,31]. The roots of *A. vulgaris* are not rich in bitter components, and therefore, they do not induce gastric secretion.

The essential oil of *A. vulgaris* is used in insect repellents and fumigants. In addition, it exhibits antibacterial and antifungal properties [3,7,18].

A monograph of the raw material *A. vulgaris herba* was prepared by the German Commission in 1988. This document listed only the traditional, above-mentioned uses of the herb and emphasized that the effectiveness of preparations based on *A. vulgaris* had not been confirmed and hence they are not recommended for therapeutic uses [51]. 

In one of the latest editions of European Pharmacopoeia [52] and the French Pharmacopoeia [53], *A. vulgaris* is listed as a homeopathic raw material. Its application includes the treatment of irregular menstrual cycles and menopausal symptoms [7], and nervous disorders such as sleepwalking, seizures, epilepsy, and anxiety [99]. The homeopathic medications are prepared using a fresh-root tincture, which is made of 65% ethanol. The tincture should contain a minimum of 0.01% (*w*/*w*) derivatives of hydroxycinnamic acid, quantified as the equivalents of chlorogenic acid [100].

In allopathy, mugwort is used mainly in the form of infusions. These are prepared by pouring boiling water over one teaspoon (about 1.2 g) of dried herb and brewing the mixture covered for about 5 min. Another form of mugwort medication used in allopathy is tinctures, which are sold all over Europe [3].

The applications of *A. vulgaris* documented in scientific works and the pharmacological activity profile of this species recommended in modern phytotherapy are presented in the below sections (Table 5).

## 11. Biological Activities of Extracts Confirmed by Scientific Research

### 11.1. Antioxidant Effect

In 2008, scientists from Cairo (Egypt) evaluated for the first time the antioxidant activity of *A. vulgaris*. They found that the aqueous extract of the herb was able to scavenge 2,2-diphenyl-1-picrylhydrazyl (DPPH) radicals at IC_50_ = 11.4 μg/mL and nitric oxide (NO) radicals at IC_50_ = 125 mg/mL in the first part of the experiment. They also estimated total phenols, flavonoids, and flavonols as 19 ± 0.16 mg/g gallic acid equivalents and 7.96 ± 0.76 and 3.4 ± 0.0 mg/g rutoside equivalents, respectively. In the second part of the experiment, they observed a significant increase in the levels of ascorbic acid and glutathione, and an increase in the activity of superoxide dismutase in the blood of rats administered with the extract at a dose of 100 mg/kg body weight (BW). The results of the study showed that *A. vulgaris* exhibits antioxidant activity and can thus be helpful to treat oxidative stress-related diseases [8].

Some newer investigations documented the antioxidant activity of the whole plant [101], its aerial parts [102] and leaf extracts [59], and its essential oil [120].

Various modern techniques have been applied in the above studies. Oyedemi and Coopoosamy proved the strong antioxidant potential of the extracts of *A. vulgaris* obtained from South Africa based on lipid peroxidation, protein glycation, xanthine oxidase, and the sTable DPPH radical scavenging assays [101]. Ben Nasr et al. investigated the antioxidant potential of the aqueous extracts of Tunisian *A. vulgaris* using the following in vitro techniques: ABTS (2,2′-azino-bis(3-ethylbenzothiazoline-6-sulfonic acid assay), DPPH, hydroxyl, superoxide, and NO scavenging assays, ferric reducing power activity assay (for determining total antioxidant capacity), and thiobarbituric acid reactive species assay (for determining the inhibition of lipid peroxidation) [103].

### 11.2. Hypolipemic Effect

An extract obtained from the root of *A. vulgaris* was tested by a Hindu team. They analyzed the rats in which hyperlipidemia had been caused by the administration of a high-fat diet for a period of 30 days. In the following month, the rodents received a standard diet. The team found that the *A. vulgaris* root extract showed a significant lipid profile-normalizing activity. Total cholesterol (TC) was reduced to 180 mg/dL, triglycerides (TG) to 147.2 mg/dL, low-density lipoprotein (LDL) cholesterol to 126.3 mg/dL, and very low-density lipoprotein (VLDL) cholesterol to 28.2 mg/dL, while the level of high-density lipoprotein (HDL) cholesterol and atherogenicity indicator (AI) increased to 68 and 2.63 mg/dL, respectively, compared to the control group (TC = 282.23 mg/dL, TG = 243.2 mg/dL, LDL cholesterol = 209.16 mg/dL, VLDL cholesterol = 47.56 mg/dL, HDL cholesterol = 34.17 mg/dL, and AI = 8.2 mg/dL) [9].

The hypolipidemic and anti-inflammatory effects of the *A. vulgaris* extract were studied in hypercholesterolemic rats by a Chinese team. They induced hypercholesterolemia in rats by feeding them with a high-fat diet containing 3% cholesterol in olein oil, for 8 weeks. This led to a significant increase in the serum levels of triglycerides, TC, LDL cholesterol, malondialdehyde, NO, and tumor necrosis factor-α and a significant decrease in the serum level of HDL cholesterol, activity of hydroxymethylglutaryl-CoA reductase in the liver, and activity of paraoxonase-1 as compared to the normal control group. Treatment of rats with *A. vulgaris* extract at a dose of 100 mg/kg per day for 4 weeks normalized the serum lipid profile, significantly increased the paraoxonase-1 activity, and decreased the serum levels of malondialdehyde, NO, and tumor necrosis factor-α as compared to the high-fat diet-treated animals. Moreover, the extract caused a decrease in the activity of hydroxymethylglutaryl-CoA reductase as compared to both high-fat diet-treated animals and control ones [104].

### 11.3. Hepatoprotective Effect

In 2005, two teams from Pakistani universities in Karachi investigated the hepatoprotective properties of *A. vulgaris* herb extracts in in vivo studies on mice. They divided the test rodents into five groups and conducted an experiment. The first and second groups were administered with a saline solution; two hours later, the second group received an additional 700 mg/kg d-galactosamine and 1 μg/kg lipopolysaccharide, which caused liver inflammation as confirmed by elevated levels of hepatic function indicators—alanine aminotransferase (ALAT) and aspartate aminotransferase (ASPAT). The third, fourth, and fifth groups were given different, prophylactic, doses (from 150 to 600 mg/kg BW) of a crude hydromethanolic extract prepared from the aerial parts of *A. vulgaris*, and an hour later, received d-galactosamine and lipopolysaccharide. A significant reduction was noted in ASPAT and ALAT activities in plasma in all the three groups. The results observed in these groups were confirmed by a histopathological examination of the liver, which showed a decrease in cellular edema and apoptotic cell count, and no hyperemia of the hepatic parenchyma, relative to the second group [105].

### 11.4. Antispasmodic Effect

A group of researchers in Cardiff (UK) tested the extracts prepared from the herb of *A. vulgaris* for their activity toward biogenic amine receptors in the smooth muscles of the gastrointestinal tract and respiratory tract of guinea pigs. The antagonistic effects of chloroform and methanol extracts were determined using concentration–response curves for contractions of the trachea and the small intestine under the influence of 5-hydroxytryptamine, methacholine, histamine, and phenylethylamine. The team found that both extracts had an antagonistic effect on the H1 receptors and caused the relaxation of smooth muscles. Two main compounds were isolated from the chloroform fraction—yomogin and 1,2,3,4-diepoxy-11(13)-eudesmen-12,8-olide—of which the former showed significant antagonism toward the H1 receptor in the ileum [10].

Researchers in São Paulo (Brazil) studied the antinociceptive activity of *A. vulgaris* herb extracts and observed their antispasmodic effects. They demonstrated that in mice treated with 500 or 1000 mg/kg hydroethanolic extract, a 48% or 59% inhibition of abdominal contractions was caused by the acetic acid solution, respectively [11].

### 11.5. Bronchodilatory Effect

Khan and Gilani tested the antispasmodic, bronchodilatory, tracheorelaxant, and antidiarrheal activities of the crude extract of *A. vulgaris* in the isolated tissue preparations of rabbit jejunum and guinea pig trachea, and also analyzed the in vivo castor oil-induced bronchodilation. They found that the *A. vulgaris* extract exhibited a combination of anticholinergic and Ca^2+^ antagonist mechanisms, which seemed pharmacologically promising for the treatment of airways disorders. The authors supposed that alkaloids, coumarins, flavonoids, saponins, sterols, tannins, and terpenes are the compounds responsible for the concentration-dependent (0.03–10 mg/mL) broncholytic effect of the *A. vulgaris* extract [106].

Natividad et al. proved that the Philippines *A. vulgaris* medicinal plant exhibited antagonistic activity at selected biogenic amine receptors in the smooth muscles of the airways and gastrointestinal tract. The chloroform and methanol extracts of the plant showed histamine H1 antagonism in the ileum and trachea. The researchers indicated that yomogin (sesquiterpenoid lactone) was the compound responsible for these activities [10]. 

### 11.6. Analgesic Effect

The team from São Paulo (Brazil) also investigated the antinociceptive properties of a hydroalcoholic extract prepared from the aerial parts of *A. vulgaris* using a mice model. Two tests were performed by the researchers: a hot plate test to measure the central analgesic effect and a writhing test to measure the peripheral analgesic effect. In the hot plate test, the control group was given only water, while the test group received 500 or 1000 mg/kg of *A. vulgaris* extract. Mice given 20 mg/kg BW morphine were adopted as the positive control. The response time (response latency) was determined after placing the rodents on a plate heated to 55 °C. The time taken by the mice to respond was not found to decrease after the administration of the *A. vulgaris* extract. In the writhing test, the mice were divided into groups and were given water, 20 g/kg BW morphine, and the hydroalcoholic extract of *A. vulgaris* at doses of 100, 250, 500, and 1000 mg/kg BW. After 30 min, the rodents were administered with a 0.8% acetic acid solution and writhing episodes were determined for the next 10 min. No decrease in the number of episodes was observed with the extract administered at the doses of 100 and 250 mg/kg BW; however, a decrease was observed with the higher doses of the extract (i.e., 500 and 1000 mg/kg BW). The results proved that the tested extracts of the *A. vulgaris* herb had a moderate peripheral antinociceptive effect but not a central effect [11].

### 11.7. MAO Inhibitory Effect

Lee et al. (South Korea) isolated flavonoids (jaceosidine, eupafolin, luteolin, quercetin, apigenine) and coumarins (esculetin, esculetin-6-methylether, scopoletin) from 80% aqueous ethanol extracts of whole *A. vulgaris* plants, and indicated that these compounds are good inhibitors of mouse brain monoamine oxidase (MAO) enzyme. The compounds showed MAO inhibitory effects at the IC_50_ values of 19.0, 25.0, 18.5, 12.5, 1.0, 31.1, 32.2, and 45.0 µmol, respectively [107].

### 11.8. Antihypertensive Effect

The aqueous and chloroform extracts of the aerial parts of *A. vulgaris* were tested by two teams from the Philippines to determine the hemodynamic potential of the plant. They found that the administration of a 10% solution of the aqueous extract into the isolated perfused mesentery of rats effectively reversed the hypertensive effect induced by noradrenaline; however, the baseline blood pressure values and heart rate remained unaltered [12].

The Chinese team performed a systematic review of randomized controlled trials based on a review of articles published from 1980 to 2013 in databases (CENTRAL, Pubmed, CBM, CNKI, VIP, and online clinical trial registry websites). They exhibit that in randomized controlled trials, moxibustion (a traditional Chinese method that uses the heat generated by burning herbal preparations containing *A. vulgaris* to stimulate acupuncture points) showed lowering of the blood pressure compared to antihypertensive drugs by stimulation of acupoint KI 1. Meta-analysis showed superior effects of moxibustion plus antihypertensive drugs (like: Metoprolol, Nifedipine, Enalapril) on systolic blood pressure (WMD (weighted mean difference): −4.91 [−7.54, −2.28]) but no superior effects on diastolic blood pressure (WMD: −6.38 [−17.17, 4.41]) [108].

### 11.9. Estrogenic Effect

The flavonoids present in the aerial parts of *A. vulgaris* were evaluated for estrogenic activity by three scientific institutions from USA and South Korea in cooperation. *Saccharomyces cerevisiae* fungi containing an expression plasmid with the cDNA of the human estrogen receptor and a reporter plasmid with the α-galactosidase gene was used in the experiment. The estrogenic effect, 5% relative to 17-β-estradiol, was observed in the polar extract (extraction with ethyl acetate), whereas the less polar ethanolic extract showed no activity. After testing the individual flavonoids of the plant, it was observed that the transcription of the reporter gene was significantly induced by eriodictyol and apigenin. The activity was also found to be concentration-dependent [12].

Shaik et al. (India) confirmed the strong anti-implantation and estrogenic activities of the leaf extracts from *A. vulgaris* on female Wistar rats [109].

### 11.10. Cytotoxic Effect

A methanolic extract prepared from the aerial parts of *A. vulgaris* was tested by Turkish teams to analyze its cytotoxic effects on human cancer cell lines (MCF7—estrogen-dependent breast adenocarcinoma cell line, A549—non-small cell lung cancer cell line, HeLa—cervical cancer cell line), and normal cell lines (A7R5—vascular smooth muscle cell line, 293T—human embryonic kidney cell line transformed with SV40 large T antigen). Cytotoxic activity was assessed by real-time electrical impedance measurements. A statistically significant inhibitory effect was observed against MCF7 (IC_50_ = 190 ng/mL), HeLa (IC_50_ = 284 ng/mL), A7R5 (IC_50_ = 382 ng/mL), and 293T (IC_50_ = 317 ng/mL), whereas a weak influence was found on A549 (IC_50_ = 778 ng/mL) cells [14]. Saleh et al. from Saudi Arabia evaluated the toxicity of the essential oil extracted from the buds and leaves of the *A. vulgaris* plant, and characterized its growth inhibitory effects on cancer cells. The results demonstrated that the essential oil-induced apoptosis in HL-60 leukemic cell line was mediated by caspase-dependent pathways, involving caspase-3, -9, and -8, which were initiated by Bcl-2/Bax/Bid-dependent loss of mitochondrial membrane potential, leading to the release of cytochrome c to the cytoplasm to activate the caspase cascade. The results showed that the studied essential oils were more efficient in inducing apoptosis in different cancer cell lines than noncancerous cells. Based on these observations, the authors suggested that *A. vulgaris* might be a promising source of new anticancer agents [111].

The latest study from 2020 was performed by a Serbian team on methanolic extracts of the *A. vulgaris* plant. The researchers proved the genotoxic and cytotoxic activities of the extracts, both separately and in co-treatment with a known mutagen (mitomycin C). They used the cytokinesis-block micronucleus assay for measuring the micronucleus frequency in human peripheral blood lymphocytes and MTT assay as the proliferation test in SW-480 human colon cancer cells and human periodontal ligament stem cells used as control. The results of the cytokinesis-block micronucleus assay showed that both extracts significantly increased the micronucleus frequency in the peripheral blood lymphocytes treated with the *A. vulgaris* extract at all the tested concentrations (10, 50, 100, and 250 μg/mL), except the lowest one (10 μg/mL). The extracts induced cytotoxic activity only in co-treatment with mitomycin C after long-term exposure and did not significantly affect the viability of the human periodontal ligament stem cells. The researchers indicated that these activities were induced by the flavonoids and other phenolic compounds present in the plant [110].

### 11.11. Antifungal and Antibacterial Activities

Researchers from Romania investigated the antifungal activity of a commercial preparation of the essential oil extracted from the *A. vulgaris* herb against *Candida albicans*. The disk-diffusion method was used in the in vitro experiment. The observed inhibition zone measured 12.5 mm. The zone of the positive control, carried out with nystatin, measured 15.0 mm, while that of the negative control, carried out with an empty paper disc, measured 6.0 mm. The results thus confirmed the antifungal activity of the essential oil of the herb against *C. albicans* [15].

In 2006, Blagojević et al. from Serbia and Montenegro also studied the effects of *A. vulgaris* essential oil on various microorganisms. The oil was isolated from the aerial and underground parts of the plant by steam distillation. After 10- and 30-fold dilutions, the zone of inhibition of pathogen growth on paper filters was examined. The oil extracted from the aerial parts exhibited inhibitory activity against various bacteria (*Escherichia coli*, *Salmonella enteritidis*, *Pseudomonas aeruginosa*, *Klebsiella pneumoniae*, and *Staphylococcus aureus*) and fungi (*C. albicans* and *Aspergillus niger*), which was attributed to its high levels of 1,8-cineole and β-thujone. On the other hand, the oil extracted from the underground parts of the plant exhibited only a low activity against the listed pathogens, due to the low level of 1,8-cineole and the lack of β-thujone in the roots [16].

In another experiment, an aqueous leaf extract of *A. vulgaris* was found to exhibit antibacterial activity against the C and D serotypes of *Streptococcus mutans* [3].

Some recent studies also documented the antimicrobial activity of the whole plant and leaf extracts [102,113,121] as well as the essential oil of *A. vulgaris* [67,112].

### 11.12. Anti-Inflammatory Activity 

El-Tantawy from Pakistan tested the anti-inflammatory and hypolipidemic effects of *A. vulgaris* extract in hypercholesterolemic rats. Hypercholesterolemia was induced in the rats by feeding them with a high-fat diet containing 3% cholesterol in olein oil, for 8 weeks. Treatment of these rats with *A. vulgaris* extract at a dose of 100 mg/kg per day for 4 weeks normalized the serum lipid profile, significantly increased the paraoxonase-1 activity, and decreased the serum levels of malondialdehyde, NO, and tumor necrosis factor-α as compared to the animals that received only high-fat diet. Moreover, the extract caused a significant decrease in the activity of hydroxymethylglutaryl-CoA reductase as compared to both high-fat diet-treated animals and control ones [104].

Afsar et al. from India studied the anti-inflammatory activity of a methanolic extract of *A. vulgaris* leaves using the cotton pellet granuloma method. The *A. vulgaris* extract was administered to rats (150–250 g BW) at doses of 200 and 400 mg/kg BW, respectively, after surgical insertion of cotton pellets into their groin region. The study was conducted following the guidelines of the “cotton pellet granuloma method.” The extract showed anti-inflammatory activity at both 200 and 400 mg/kg, whereas the results were more significant at 400 mg/kg compared with the control [115].

The latest study performed on the extracts of Tunisian *A. vulgaris* plants proved their moderate anti-inflammatory activity based on lipoxygenase inhibition assay [103].

### 11.13. Antiallergenic Effect

Olsen et al. from Denmark studied the specificity and efficacy of immunotherapy with *A. vulgaris* extracts on 25 patients having seasonal rhinoconjunctivitis for more than two years and only two clinically important allergies—either to *A. vulgaris* and *Betula verrucosa* or to *A. vulgaris* and *Phleum pratense*. Twenty patients completed two years of specific immunotherapy. Nine patients were treated with the extracts of *A. vulgaris* and 11 with the extracts of either *B. verrucosa* or *P. pratense*. Treatment with *A. vulgaris* was followed by a significant decrease in skin and eye sensitivity to *A. vulgaris* but not to *B. verrucosa* or *P. pratense.* No significant decrease was observed in medicine consumption or symptom scores. The patients treated with *B. verrucosa* or *P. pratense* experienced a significant decrease in skin and eye sensitivity to these organisms, but not to *A. vulgaris*, and showed a significant decrease in medicine consumption and symptom scores in the *B. verrucosa* or *P. pratense* season but not in the *A. vulgaris* season. The treatment was both effective and specific, with one unexplained exception that both patient groups (*A. vulgaris* and *B. verrucosa* or *P. pratense*) experienced decreased skin sensitivity to *A. vulgaris* [116].

### 11.14. Antimalarial Activity 

Kodippili et al. from Sri Lanka investigated the antimalarial (both antiparasitic and antidisease) activity of the *A. vulgaris* extract. They assessed the antiparasitic activity of the extract at three doses (250, 500, and 1000 mg/kg) in vivo in the *Plasmodium yoelii* rodent malaria model, using distilled water as the negative control and Coartem as the positive control. In the 4-day suppressive assay, the oral administration of the extract at 500 and 1000 mg/kg significantly inhibited parasitemia by 65.16% and 51.46%, respectively. The antinociceptive activity of the extract was analyzed using the hot plate test, which indicated a central, supraspinally mediated response in relieving pain. The antidisease activity of the extract was further corroborated by the increased survival of the infected mice treated with the 500 mg/kg dose. The *A. vulgaris* extract was well tolerated by the mice over a period of 14 days (assay of subchronic toxicity), with no overt signs of toxicity or stress. Hepatotoxicity (evaluated in terms of the serum levels of glutamic–oxaloacetic transaminase and serum glutamic–pyruvic transaminase), renotoxicity (in terms of serum urea and creatinine), and hematotoxicity (in terms of total RBC, WBC, and differential leukocyte counts) were also ruled out. Based on these findings, the authors concluded that *A. vulgaris* leaf extract is orally active and nontoxic, and that *A. vulgaris* is a weed with the potential to act as a cheap source of the antimalarial plant [117].

Another team from the same center from Sri Lanka studied the ethanolic leaf extract of *A. vulgaris* for antiparasitic activity in *Plasmodium berghei* ANKA murine malaria model that elicits similar pathogenesis as falciparum malaria. The extract at doses of 500, 750, and 1000 mg/kg significantly inhibited parasitemia by 79.3%, 79.6%, and 87.3% respectively, in the 4-day suppressive assay. Chronic administration of the extract at high dose ruled out the overt signs of toxicity and stress as well as hepatotoxicity, renotoxicity, and hematotoxicity. Therefore, the authors claimed that the oral administration of a crude extract of *A. vulgaris* is nontoxic and induces antimalarial (antiparasitic) effects [118].

### 11.15. Anthelmintic Activity

Caner et al. from Turkey proved that the extracts of *A. vulgaris* and *A. absinthium* induce anthelmintic effects against trichinellosis (*Trichinella spiralis*) in rats. The results of trichinoscopy and artificial digestion showed that during the enteral (adult) phase of the illness, 300 mg/kg of methanol extracts prepared from the aerial parts of *A. vulgaris* and *A. absinthium* reduced the larval rate by 75.6% and 63.5% in the tongue, 53.4% and 37.7% in the diaphragm, 67.8% and 46.2% in the quadriceps, and 66.7% and 60.5% in the biceps–triceps muscles of rats, respectively. Furthermore, during the parenteral (encapsulated larvae) phase, 600 mg/kg dose of both the plant extracts decreased the larval rate by 66.4% and 59.9% in the tongue, 57.4% and 50.0% in the diaphragm, 47.6% and 43.7% in the quadriceps, and 60.2% and 46.4% in the biceps–triceps muscles of rats, respectively. In addition, antibody analysis showed that *A. vulgaris* extract can significantly reduce the antibody response [119].

### 11.16. Insecticidal Activity

The insecticidal activity of *A. vulgaris* was studied by a Hindu team from Tiruchirappalli in India. They exposed the larvae of *Aedes aegypti* (Egyptian mosquito, dengue virus vector) to various concentrations of the essential oil extracted from the *A. vulgaris* herb. Even at a low concentration of 10 ppm, insecticidal activity was observed after 24 h, while the best results were seen at a concentration of 500 ppm, where after 8 h of exposure to the oil solution, the larvae mortality rate was 100%. Thus, the study showed that the essential oil of *A. vulgaris* might serve as a potential insecticide [17].

The activity of essential oil obtained from the leaves and nanoparticles prepared with leaf extracts was evaluated by Lavor et al. from Brazil and Balasubramani et al. from India [122,123].

Furthermore, other teams have documented the effect of different raw materials against another insect species—*Culex quinquefasciatus* [124] and some stored product insects [125] (Table 6).

## 12. Applications in Cosmetology

Based on its profile of activity, the European database of cosmetic raw materials, CosIng (Cosmetic Ingredients database), recommends the use of *A. vulgaris* in eight forms, including skincare agents, humectants, skin protectants, and fragrances. Original forms used in cosmetics are filtrates obtained as a result of fermentation by bacteria (*Bacillus* sp., *Lactobacillus* sp.) or fungi (*Saccharomyces* sp.) (Table 7).

During fermentation, *Bacillus* sp. produce valuable physiologically active substances such as peptides, viscous compounds (with polysaccharide structure), antioxidants, and fibrin.

A combination of *A. vulgaris* and *Bacillus* sp. has been shown to enhance the effects of fermentation and increase the antiaging and antiwrinkle effects by inhibiting the production of matrix metalloproteinase-1 and -9 enzymes (decomposed of collagen) and increasing cell regeneration and collagen synthesis [127].

In cosmetics production, the extracts of herb and essential oil are used, which are the raw materials that do not contain pollen causing allergic reaction. 

Cosmetic products containing *A. vulgaris* extracts are available on the European, Asian, and American markets. The species is of particular interest to South Korean companies such as *Hanyul*, *Missha*, *Isntree*, *I’m From*, *Round lab*, *VelyVely*, *Dr. Jart+*, and *Tonymoly*. They produce cosmetics (mainly in the form of creams) using the extracts of *A. vulgaris* herb, which are intended for skincare. In Russia, *PervoeReshenie*, *Etude House*, *Natura Siberica*, and *Fitocosmetic* use *A. vulgaris* in the production of, for example, toothpastes, balms, and creams. The plant extracts are also found in the products sold by American cosmetic companies, including *Soleil Toujours*, *AoSkincare*, and *TruBaby*.

## 13. Applications in the Food Industry

The essential oil extracted from the aerial parts of *A. vulgaris* is useful in the food industry, as it exhibits antiseptic, antioxidant, and antimicrobial activities. In addition, the oil has larvicidal, nematicidal, and pesticidal effects [46].

The species *A. vulgaris* is highly valued as a spice because of the aroma and bitter taste of the herb and the sweet and spicy taste of the roots. The leaves and buds collected just before flowering are used as a bitter additive for seasoning rice dishes and tea in Asia [14,18]. The species is also used as an additive to meat, poultry, fish, and salads, in the production of vodkas and herbal wines [18], and in the preparation of sweet and savory cakes (especially in Japan). Before the introduction of hops, *A. vulgaris* had been used to flavor beer [18].

Despite its earlier popularity, *A. vulgaris* is relatively rarely used as a spice in Poland. Currently, there are attempts being made to make it more popular as a seasoning agent for, *inter alia*, mutton, liver, cabbage, spinach, and mushroom dishes, as well as soups.

In addition, *A. vulgaris* tincture is widely used in animal feed as a sensory (aromatic) additive [128].

The EFSA reports that as an insecticide *A. vulgaris* is intended for use in arable fields, for protecting plants in orchards and vineyards, and for growing vegetables. The plant targets insects such as *Ae. aegypti* (Egyptian mosquito), *Musca domestica* (housefly), and *Tribolium castaneum* (red flour beetle). There is increasing interest in using the plant for this purpose because it is less harmful to both humans and the environment compared to other preparations [18,46,129].

## 14. Safety of Use

The use of *A. vulgaris* herb extracts in therapeutic doses is not likely to cause side effects; however, the plant can cause allergies, as confirmed by the U.S. Food and Drug Administration (FDA). Its pollen contains allergenic glycoproteins that cause the type I (immediate) allergic reaction, related to IgE antibodies. In addition, anaphylactic shock has been observed in patients after swallowing the pollen [3,130]. The species is also considered to be the main cause of hay fever and allergic asthma in northern Europe, North America, and part of Asia [18,131].

If a person is allergic to any ingredient of *A. vulgaris* or any plant of the family *Asteraceae*, he/she should avoid contact with them. Cross-reactions of the plant with pollen from other plants as well as with food substances have also been observed—with birch, cabbage, grasses, hazelnuts, honey, pollen of the European olive and sweet pepper, and also with royal jelly, sunflower, kiwi, peach, mango, apple, celery, and carrot [18,132].

In addition to anaphylactic shock, *A. vulgaris* pollen, as an allergen, can cause breathing difficulties, bronchospasm, airway hypersensitivity, asthma attack, seasonal rhinitis, and conjunctivitis. Allergic skin reactions, such as dermatitis and urticaria, may also occur [92,131,133,134].

When consumed in large doses, *A. vulgaris* may cause miscarriage [51,92], nausea, vomiting, and nervous system damage. Furthermore, cases of hypertension have been reported [18].

The EFSA lists that the ingredients in the essential oil of *A. vulgaris* herb, such as α-thujone, β-thujone, camphor, and 1,8-cineole, have potentially adverse effects on human health, when taken with food or dietary supplements [135]; however, it emphasizes that most research has concerned only a more concentrated oil, and not a less concentrated extract [18].

It should be remembered that *A. vulgaris* should be used with caution in patients with diabetes, as it can increase blood glucose levels. The species is also not recommended for the prevention of malaria and in vitro studies have not shown protozoicidal activity. In addition, it was reported that patients who used *A. vulgaris* as a prophylactic agent when traveling to eastern Africa were not prevented from developing malaria [18].

The EFSA has also published a document describing the safety of using a hydroethanolic tincture of *A. vulgaris* having a dry matter content of approximately 1.7% as an animal feed additive. The ruling emphasizes that although the isolated phenolic compounds are not toxic, the safety profile of this tincture cannot be determined because as much as 74% of the dry matter fraction remains undetermined. It is also not known whether the tincture can cause irritation to the skin or eyes. The document is summarized with a statement declaring that no further research is needed because *A. vulgaris* and its extracts are commonly used as aromatic substances in food, where they perform similar functions as in feed [128].

**Figure 4:**
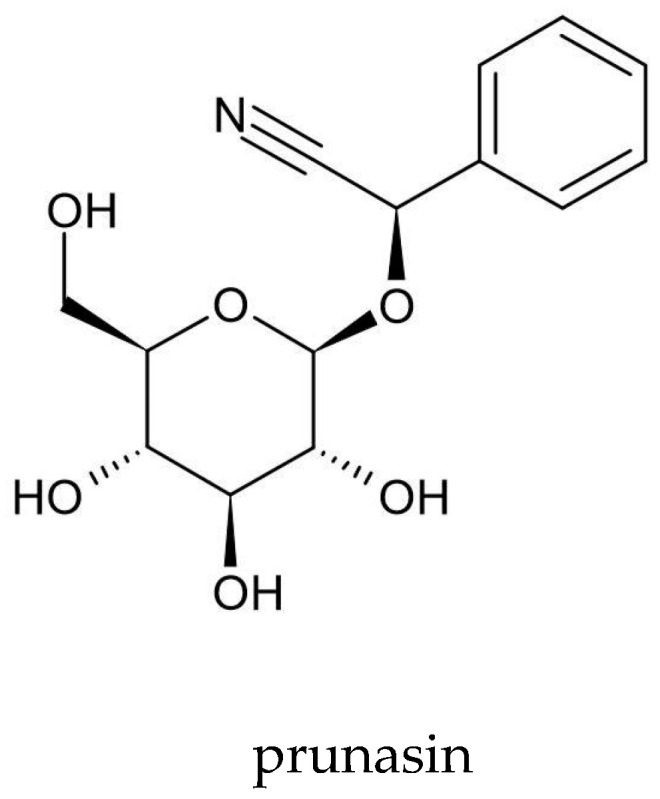
Chemical structure of prunasin—a cyanogenic glycoside isolated from *A. vulgaris.*

Scientists from four Japanese centers—Science University of Tokyo (Tokyo), The Institute of Physical and Chemical Research (RIKEN) (Wakō), Nagoya University School of Medicine (Nagoya), and Aichi Cancer Center Research Institute (Nagoya)—have isolated prunasin from *A. vulgaris* (Figure 4). They used 300 mg of acetone extract and obtained 25 mg of prunasin [63]. Prunasin is a cyanogenic glycoside that releases toxic hydrogen cyanide (HCN) during enzymatic hydrolysis by β-3-glucosidases in the macerated plant tissue or under the influence of intestinal microflora. In the human body, HCN combines with cytochrome oxidases and prevents oxygen access to tissues, thereby leading to body hypoxia [136]. However, further research is needed on the prunasin content in *A. vulgaris* and its effect on the human body.

## 15. Biotechnological Research on Micropropagation

Excessive exploitation and exposure to anthropogenic activities can cause a change in the chemical composition of *A. vulgaris*. In addition, the species rarely reproduces from seeds, which require symbiosis with soil microflora for germination. An alternative way of reproduction for the plant is micropropagation—a technique used for rapid multiplication in vitro. It provides conditions under which the progeny plant is genetically stable and its metabolites are identical in chemical composition to that of the parent plant [69].

At the University of Mysore in Karnataka (India), Sudershana et al. [44] investigated the effect of different phytohormone concentrations on the micropropagation of *A. vulgaris* in 2010. They used young leaf explants and the Murashige-Skoog (MS) medium enriched with 1 mg/l 6-benzylaminopurine (BAP) and 3 mg/L 1-naphthylacetic acid, which proved optimal for inducing callus growth. The callus was then transferred to a medium containing 1 mg/L BAP and 3.0 mg/L gibberellic acid (GA_3_), which was found to be optimal for shoot development. The addition of indolylacetic acid (IAA) at 0.5–1 mg/L induced root development [44].

In 2013, the same group of scientists developed a protocol for regenerating *A. vulgaris* from encapsulated somatic embryos (so-called somatic seeds). The plant development was observed to be best when encapsulation was done with a 2% alginate solution cured for 30 min in a 75 mM calcium chloride solution. It was proved that the encapsulated embryos of *A. vulgaris* could be stored at 4 °C, 20 °C, and 22 °C for 4 months. These embryos showed the highest (90%) efficiency of conversion into microplants at 22 °C on MS medium containing 1.5 mg/L GA_3_, 0.5 mg/L IAA, and 40 mg/L ascorbic acid [137].

An effective protocol for the micropropagation of *A. vulgaris* was developed by scientists from Bharathidasan University in Tamil Nadu (India) in cooperation with Universita di Pisa in Pisa, (Italy). The method involved in vitro liquid culturing on MS medium with the addition of BAP at concentrations from 0.44 to 8.88 μM. In 100 mL flasks, the frequency of shoot formation from explants was determined to be 99.9% when the BAP concentration was 4.44 μM. The largest number of shoots (85.5) was obtained after 30 days of culture in 500 mL flasks, with 4.44 μM BAP. An increase in shoot proliferation with an increase in flask capacity and a decrease in proliferation were observed with an increase in the BAP concentration above 4.44 μM. In the next stage of work, individual shoots were isolated and rooted on MS medium with 8.56 μM IAA. The seedlings obtained were acclimatized under standard laboratory conditions. The plants thus grown were then transferred to the greenhouse [64].

## 16. Conclusions

*Artemisia vulgaris*, a species which played a significant role in the history of European medicine, was referred to in the Middle Ages as the “mother of herbs,” and used *inter alia* in treating gynecological and urological ailments and gastrointestinal tract diseases, is not a pharmacopoeial species today in the global allopathic medicine. However, it occupies an important position in traditional medicine, both in Europe and Asian countries, mainly China and India.

Currently, *A. vulgaris* is the subject of numerous phytochemical and pharmacological studies. Phytochemical studies have proved the rich composition of the aerial parts of this plant, which consists of sesquiterpenoid lactones, flavonoids, and coumarins, and an essential oil made of qualitatively variable components. In turn, pharmacological studies have provided evidence of very valuable, previously unknown biological activities of the raw materials of *A. vulgaris*, including antioxidant, hypolipemic, hepatoprotective, antispasmodic, analgesic, antihypertensive, estrogenic, cytotoxic, antibacterial, and antifungal effects. Due to the high variability in its chemical composition, this species is also subject to biotechnological research, where attempts are being made to genotypically multiply high-production plants using micropropagation methods. A major part of this research is conducted by non-European research centers. This is because the species is widely distributed on as many as four continents—Europe, Asia, North America, and South America—and there is a slightly greater interest in this species outside of Europe.

In East Asian countries, mainly China and Japan, as well as in Europe, this species is well known as a spice. In Europe, the current interest in the species as a spice is definitely not as strong as in Asia; however, it has been increasingly used in the cosmetics industry.

The biological activities of the *A. vulgaris* raw material proven so far have raised hopes for a renaissance of the interest of contemporary medical world in the medieval “mother of herbs”.

## Figures and Tables

**Table 1:** The Latin synonymous names of *A. vulgaris* L.

*Artemisia vulgaris* Burm.f. [Illegitimate]
*Artemisia vulgaris* Mattf. [Illegitimate]
*Artemisia vulgaris* C.B.Clarke
*Artemisia vulgaris* var. *americana* Besser
*Artemisia vulgaris* f. *angustisecta* Fiori
*Artemisia vulgaris* var. *aromatica* Sacc.
*Artemisia vulgaris* subsp. *candicans* (Rydb.) H.M.Hall & Clem.
*Artemisia vulgaris* var. *candicans* (Rydb.) M.Peck
*Artemisia vulgaris* subsp. *coarctata* (Fors ex Besser) Ameljcz.
*Artemisia vulgaris* var. *coarctica* Besser
*Artemisia vulgaris* var. *douglasiana* H.St.John
*Artemisia vulgaris* subsp. *flodmanii* (Rydb.) H.M.Hall & Clem.
*Artemisia vulgaris* var. *flodmanii* (Rydb.) M.Peck
*Artemisia vulgaris* var. *gilvescens* (Miq.) Nakai
*Artemisia vulgaris* var. *glabra* Ledeb.
*Artemisia vulgaris* var. *glandulifera* (L.F.Hend.) M.Peck
*Artemisia vulgaris* subsp. *gnaphalodes* (Nutt.) H.M.Hall & Clem.
*Artemisia vulgaris* var. *gnaphalodes* (Nutt.) Kuntze
*Artemisia vulgaris* subsp. *heterophylla* (Nutt.) H.M.Hall & Clem.
*Artemisia vulgaris* var. *incana* Maxim.
*Artemisia vulgaris* var. *incanescens* Franch.
*Artemisia vulgaris* var. *incompta* (Nutt.) H.St.John
*Artemisia vulgaris* var. *indica* (Willd.) Hassk.
*Artemisia vulgaris* var. *kamtschatica* Besser
*Artemisia vulgaris* var. *kiusiana* Makino
*Artemisia vulgaris* var. *latiloba* Ledeb.
*Artemisia vulgaris* var. *leucophylla* Turcz. ex Besser
*Artemisia vulgaris* var. *littoralis* Suksd.
*Artemisia vulgaris* var. *longifolia* (Nutt.) M.Peck
*Artemisia vulgaris* subsp. *longifolia* (Nutt.) H.M.Hall & Clem.
*Artemisia vulgaris* subsp. *ludoviciana* (Nutt.) H.M.Hall & Clem.
*Artemisia vulgaris* var. *ludoviciana* (Nutt.) Kuntze
*Artemisia vulgaris* var. *maximowiczii* Nakai
*Artemisia vulgaris* var. *mexicana* (Willd. ex Spreng.) Torr. & A.Gray
*Artemisia vulgaris* subsp. *mexicana* (Willd. ex Spreng.) H.M.Hall & Clem.
*Artemisia vulgaris* subsp. *michauxiana* (Besser) H.St.John
*Artemisia vulgaris* var. *minor* Ledeb.
*Artemisia vulgaris* var. *mongolica* Fisch. ex Besser
*Artemisia vulgaris* f. *montana* Nakai
*Artemisia vulgaris* var. *nilagirica* C.B.Clarke
*Artemisia vulgaris* var. *nipponica* Nakai
*Artemisia vulgaris* f. *nipponica* Nakai
*Artemisia vulgaris* subsp. *redolens* (A.Gray) H.M.Hall & Clem.
*Artemisia vulgaris* var. *rubriflora* Turcz. ex Besser
*Artemisia vulgaris* subsp. *selengensis* Thell.
*Artemisia vulgaris* var. *selengensis* (Turcz. ex Besser) Maxim.
*Artemisia vulgaris* subsp. *serrata* (Nutt.) H.M.Hall & Clem.
*Artemisia vulgaris* var. *stolonifera* Maxim.
*Artemisia vulgaris* var. *suaveolens* Bég.
*Artemisia vulgaris* var. *tenuifolia* Turcz. ex DC.
*Artemisia vulgaris* subsp. *tilesii* (Ledeb.) H.M.Hall & Clem.
*Artemisia vulgaris* var. *umbrosa* Turcz. ex Besser
*Artemisia vulgaris* subsp. *urjanchaica* Ameljcz.
*Artemisia vulgaris* var. *verbenacea* Kom.
*Artemisia vulgaris* var. *viridissima* Kom.
*Artemisia vulgaris* var. *vulgaris*
*Artemisia vulgaris* var. *wrightii* (A.Gray) H.M.Hall & Clem.
*Artemisia vulgaris* subsp. *wrightii* (A.Gray) H.M.Hall & Clem.

**Table 2:** The chemical composition of *A. vulgaris* aerial parts.

Chemical Group	Compound	Content	References
Sesquiterpenoid lactones	1,2,3,4-diepoxy-11(13)-eudesmen- 12,8-olide; yomogin	na *	[10]
	psilostachyin, psilostachyin C	na	[3,54]
	vulgarin	0.15% d.w.	[3,30,55]
	artemisinin	0–2.3% dw.	[56,57]
Flavonoids	quercetin 3-galactoside, quercetin 3-glucoside, kaempferol 3-glucoside, kaempferol 3-rhamnoside, kaempferol 3-rutinoside, kaempferol 7-glucoside, luteolin 7-glucoside	~6 mg/kg d.w. ~11 mg/kg d.w. ~10 mg/kg d.w. ~5 mg/kg d.w. ~5 mg/kg d.w. ~5 mg/kg d.w. ~5 mg/kg d.w.	[13]
	apigenin	~6 mg/kg d.w.	[3,11,13,58]
	chrysoeriol	~2.5 mg/kg d.w.	[13]
	eriodictyol, diosmetin, isorhamnetin	~40 mg/kg d.w. ~5 mg/kg d.w. ~2.5 mg/kg d.w.	[3,13,58]
	eupafolin, homoeriodictyol	~5 mg/kg d.w. ~10 mg/kg d.w.	[13]
	hyperoside	0.5 mg/g d.w.	[3,58,59]
	jaceosidin	~3 mg/kg d.w.	[13]
	quercetin	~3 mg/kg d.w.	[3,11,30,58]
	luteolin	~40 mg/kg d.w.	[3,13,58]
	rutoside	~7–20 mg/kg d.w.	[11,13,59]
	tricine,	~3 mg/kg d.w.	[13]
	vitexin	~4 mg/kg d.w.	[3,58]
Coumarins	esculin, esculetin, umbelliferone	na	[3,60]
Phenolic acids	1,5-di-*O*-caffeoylquinic acid, 3,5-di-*O*-caffeoylquinic acid	0.3% d.w. 0.2% d.w.	[61]
	5-feruloylquinic acid quinic acid protocatechuic acid glucoside 3-*O*-caffeoylquinic acid 5-*O*-caffeoylquinic acid 4,5-*O*-di-caffeoylquinic acid	0.37 mg/g d.w. 1.5 mg/g d.w. 3.2 mg/g d.w. 0.44 mg/g d.w. 2.8 mg/g d.w. 11 mg/g d.w.	[59]
	caffeic acid	na	[3,11,61]
Sterols	sitosterol, stigmasterol		[3]
Fatty acids	na	13.3 mg/g f.w.	[46]
Carotenoids	(*E*)-β-ionone	na	[3,62]
Cyanogenic glycosides	prunasin	na	[3,63]
Vitamins	ascorbic acid	na	[30]
Polyacetylenes	na	na	[3,60]
Tannins	na	na	[11]

* na—no data available.

**Table 3:** The chemical composition of essential oil from *A. vulgaris* aerial parts.

Chemical Groups/Compounds	Estimated Content (%)	References
**Monoterpenoids**		
artemisia alcohol, artemisyl acetate, isobornyl acetate, (*Z*)-β-ocymen, terpinolene	0–2.6	[62]
artemisia ketone	0–2.89	[62,64,65]
borneol	0.4–9.8	[3,15,17,24,62,64,65,66,67]
bornyl acetate	0–6.29	[15,16,17,65,67]
camphene	1.8–9.1	[15,16,17,46,62,64,65]
camphor	0–47.7	[16,17,18,24,46,62,64,65,66,68,69]
carvone	0–0.38	[65]
trans-carveol, trans-pinocarveol	0–0.77	[15,64,65]
1,8-cineol	2.6–17.6	[3,16,17,18,24,30,46,62,64,66,70]
cis-chrysanthenol, dehydrosabinaketone, methyleugenol, verbenyl acetate, *p*-cymene-8-ol, piperitone, *p*-mentha-1,4-dien-7-ol, sabinaketone, trans-verbenol, cuminol	0–7.0	[15]
cymene	0–1.14	[17,62,65]
isoborneol	0.3–8.2	[17,62,64]
isobornyl 2-methylbutyrate, menthol	0–5	[46]
iso-3-thujanol	0–1.4	[62,64,65]
limonene	0–0.46	[65]
(*E*)-β-ocymen	0.5–2.7	[62]
3-thujanol		[17,64]
4-terpineol	0–1.4	[3,15,16,24,62,64,66]
cis-thujone	0–12.9	[46,62]
linalool	0–0.4	[3,62,64,66]
chrysanthenyl acetate	0–23.6	[15,46,62]
β-myrcene	0.1–8.8	[16,62]
sabinene	0–0.67	[15,16,17,62,64,65]
cis-sabinene hydrate,	0–1.08	[62,64,65]
trans-sabinene hydrate	0–0.55	[15,62,64,65]
santolina triene	0–0.6	[15,16,17,62,64]
α-thujone	0–3.18	[15,16,17,24,46,64,71]
β-thujone	0–1.19	[15,16,17,24,46,64,68,71,72]
α-fenchen		[16,17,64]
α-pinene	0–0.9	[15,16,46,62,64,65]
α-terpinene	0–0.4	[62,64]
α-terpineol	0–1.6	[15,24,62]
α-thujene	0.2–4.1	[15,16,62]
β-pinene	0.1–12.9	[15,17,24,46,62,64]
γ-terpinene	0–0.54	[16,62,64,65]
thymol	0–0.39	[65]
**Sesquiterpenoids**		
aromadendrene	0–0.2	[16,64]
bicyclogermacrene	0.9–2.2	[16,17,62,64]
α-cadinol	0–1.99	[3,17,66,67]
caryophyllene	0–37.45	[24,46,62,67]
caryophyllene oxide	1.52–5.5	[3,15,16,17,46,62,64,65,66]
trans-caryophyllene, trans-salvene	2.5–12.2	[46,65]
caryophylla-4(14),8(15)-diene-5-α-ol, (*E*)-nerolidol, humulene epoxide II, germacrene d-4-ol, ledol, farnesyl acetate, lanceol acetate, salvial-4(14)-en-1-one, silphiperfol-5-en-3-ol (*Z*)- β-farnesene, α-calacorene, β-chamigrene, β-longipinene	0–0.5	[62,67]
α-copaen	0–1.0	[15,16,17,62,64]
cubebene	0–12	[3]
davanone, silphiperfol-4,7(14)-diene, β-burbonen	0–0.15	[62,64]
β-elemene	0–8	[17], [64]
β-eudesmol	0–8.95	[16,46,64,65]
α-elemene, β-bisabolene	0–8.8	[16,62]
farnesene	0–0.88	[15,65]
germacrene D	5.3–15.1	[15,46,62,64]
α-humulene	0.2–8.8	[16,62,64,65]
epi-α-muurolol	0.4–1.4	[15,62]
spathulenol	1–2.5	[15,17,46,62]
7-α-silphiperfol-5-ene, epi-β-santalene, modhephene, petasitene, presilphiperfol-7-ene, silphin-1-ene, valeranone, humulene oxide, α-bisabololene, α-cedrene, α-isocomene, α-trans-bergamotene	0–0.5	[16]
**Diterpenoids**		
phytol	0–2.94	[62,67]
γ-terpineol	0–1.44	[65]

**Table 4:** Differences in the composition of essential oils from the herb of *A. vulgaris* of various origins.

Origin of Plants	Main Components	References
Brazil	caryophyllene (37.45%) germacrene D (16.17%) humulene (13.66%) borneol (6.80%) caryophyllene oxide (5.67%)	[67]
France	camphor (1–13%) 1,8-cineole (1–23%) terpinen-4-ol (1–19%)	[73]
Germany	sabinene (16%) myrcene (14%) 1,8-cineole (10%)	[74]
India	α-thujone and thujone isomer (β-thujone) camphor	[75]
Italy	camphor (47%)	[76]
	camphor (2–20%) myrcene (9–70%) 1,8-cineole (1–27%) borneol (3–18%)	[77]
Lithuania (North part)	sabinene (0–8.4%) β-pinene (0.1–12.9%) 1,8-cineole (2.6–17.6%) cis-thujone (0–12.9%) trans-thujone (0–20.2%) chrysanthenyl acetate (0–23.6%) caryophyllene (2.5–12.2%) germacrene D (5.3–15.1%)	[62]
Morocco	thujone/isothujone camphor	[78]
Vietnam	β-caryophyllene (24%) β-cubebene (12%) β-elemene (6%)	[79]
	1,8-cineole camphor α-terpineol	[80]

**Table 5:** Pharmacological properties of *A. vulgaris* herb and root extract.

Activity *	Information	Compounds Supposed to be Responsible	References
Antioxidant	Proved by different methods: DPPH, lipid peroxidation, protein glycation, xanthine oxidases, ABTS, hydroxyl superoxide, nitric oxide, ferric reducing power activity and inhibition of lipid peroxidation by thiobarbituric acid reactive species assays. Increasing the level of ascorbic acid and glutathione.	flavonoids, flavonols, phenolic acids	[8,59,101,102,103]
Hypolipemic	Normalized serum lipid profile, a significant increase in paraoxonase-1 activity and decrease in serum malondialdehyde, nitric oxide, tumor necrosis factor-α level and decrease in hydroxymethylglutaryl-CoA reductase activity. Lowering total cholesterol, triglycerides, LDL, VLDL, and increasing HDL and atherogenicity indicator (aqueous extract of *A. vulgaris* roots) *.		[9,104]
Hepatoprotective	Prophylactic protective effect limiting inflammation, cellular oedema, apoptotic cell count, and hyperaemia of the hepatic parenchyma.		[105]
Antispasmolytic	Antagonism towards H1 histamine receptors.		[10,11]
Bronchodilatory	Anticholinergic and Ca^2+^ antagonist mechanisms. Histamine H1 antagonism in the ileum and trachea.	yomogin (sesquiterpene lactone), alkaloids, coumarins, flavonoids, saponins, sterols, tannins, terpenoids	[10,106]
Analgesic	Mild peripheral anti-nociceptive effect.	probably induced by rutoside, hydroxybenzoic acid derivatives, and caffeic acid and its derivatives	[11]
MAO inhibition	Inhibition of mouse brain monoamine oxidase (MAO) enzyme.	flavonoids: jaceosidine, eupafolin, luteolin, quercetin, apigenine; coumarins: esculetin, esculetin-6-methylether, scopoletin	[107]
Antihypertensive	Inhibiting the hypertensive effect of noradrenaline. Moxibustion showed lowering the blood pressure compared to antihypertensive drugs by stimulation of acupoint KI 1.	na ** Moxibustion—a traditional Chinese method that uses the heat generated by burning herbal preparations containing *A. vulgaris* to stimulate acupuncture points	[12,108]
Estrogenic	Antagonism towards the estrogen receptor and activation of gene transcription. Induction of gene transcription by eriodictyol and apigenin. Anti-implantation activity and estrogenic activity on female Wistar rats.	flavonoids	[13,109]
Cytotoxic	Inhibition of tumour cell growth in cancer cell lines: MCF7, HeLa, A7R5, 293T, HL-60 and SW-480.	phenolic compounds, flavonoids, essential oil	[14,110,111]
Antifungal and antibacterial	Inhibitory effect of the oil fraction on the development of *Candida albicans*. Inhibitory effect of the oil fraction on the development of *Escherichia coli, Salmonella enteritidis, Pseudomonas aeruginosa, Klebsiella pneumoniae, Staphylococcus aureus, Streptococcus mutans, Candida albicans,* and *Aspergillus niger.*	probably associated with the presence of essential oils, 1,8-cineole, α-thujone and camphene	[15,16,67,102,112,113,114]
Anti-inflammatory	Normalization of serum lipid profile, increase in paraoxonase-1 activity and decrease in serum malondialdehyde, nitric oxide and tumor necrosis factor-α level. Proved by lipoxygenase (LOX) inhibitory activity assay and “Cotton Pellet Granuloma Method”.	na	[103,104,115]
Antialergenic	Decrease in skin sensitivity and eye sensitivity.	na	[116]
Antimalarial	Activity angainst *Plasmodium yoelii* and *P. berghei.*	na	[117,118]
Anthelmintic	Activity against *Trichinella spiralis.*	na	[119]

* all of the listed activities have been proved for extracts of *A. vulgaris* herb, except for hypolipemic effects; ** na—no data available.

**Table 6:** Insecticidal activity of different raw materials from *A. vulgaris*—examples.

Insect Species	Pathogenity	Raw Material of *A. Vulgaris*	References
*Aedes aegypti (Egyptian mosquito)*	denge fever virus vector	essential oil from the leaves	[122,123]
		essential oil from the herb	[17]
		nanoparticles with leaf extract	[123]
*Culex quinquefasciatus* Say. *(Culex fatigans)* (arbo virus)	vector of avian malaria, vector of *wuchereria bancrofti*	leaves extracts	[124]
		stem extracts	
		root extracts	
*Tribolium castaneum* (Herbst)	stored–product insect pests	essential oil from leaves	[126]
		essential oil from aerial parts	[125]
*Callosobruchus maculatus* (F.)	stored–product beetles	essential oil from aerial parts	[125]
*Rhyzopertha dominica*	stored–product beetles	essential oil from aerial parts	[125]

**Table 7:** Applications of *A. vulgaris* in cosmetology as recommended by the CosIng database.

Name in CosIng Database	Functions
*Artemisia vulgaris* extract	skin conditioning
*Artemisia vulgaris* herb extract	perfuming
*Artemisia vulgaris* oil	perfuming, skin conditioning
*Bacillus/ Artemisia vulgaris* extract/ferment filtrate	skin conditioning
hydrolyzed *Artemisia vulgaris* leaf	humectant, skin conditioning
*Lactobacillus/Chrysanthemum zawadskii* flower/*Gleditsia japonica* fruit/*Thuja orientalis* leaf/*Morus alba* bark/*Panax ginseng* root/*Artemisia vulgaris* extract ferment filtrate	skin conditioning
*Bacillus/Cinnamomum cassia* bark/*Momordica charantia/Opuntia humifusa* fruit/*Aloevera/Artemisia vulgaris/Camellia sinensis/Nelum bonucifera* leaf/*Pueraria thunbergiana* root ferment filtrate	antioxidant, skin protecting
*Saccharomyces*/*Aloe barbadensis* leaf/*Artemisia vulgaris* leaf/*Prunus mume* fruit ferment filtrate	skin conditioning
